# Clinical and Wavefront Outcomes After Femtosecond Laser Versus Mechanical Microkeratome Lasik: A Prospective Paired-Eye Comparative Study

**DOI:** 10.3390/bioengineering13060685

**Published:** 2026-06-14

**Authors:** Sophie-Charlotte Drogge, Andreas J. Kreis, Ivo Guber, Valentin Pajic, Vladimir Canadanovic, Zeljka Cvejic, Martina Kropp, Gabriele Thumann, Eline De Clerck, Mirko Resan, Bogdan Resan, Bojan Pajic

**Affiliations:** 1 Eye Clinic ORASIS, Swiss Eye Research Foundation, 5734 Reinach, Switzerland; sophie.charlotte.drogge@gmail.com; 2 Division of Ophthalmology, Department of Clinical Neurosciences, Geneva University Hospitals, 1205 Geneva, Switzerlandivo.guber@augenchirurgie.ch (I.G.); martina.kropp@unige.ch (M.K.); gabriele.thumann@hug.ch (G.T.); eline.declerck@hug.ch (E.D.C.); 3 Experimental Ophthalmology, University of Geneva, 1205 Geneva, Switzerland; 4 Medical Faculty, Semmelweis University, 1094 Budapest, Hungary; valentin.pajic@stud.semmelweis.hu; 5 Medical Faculty, University of Novi Sad, 21000 Novi Sad, Serbia; canadanovic.vladimir@gmail.com; 6 Department of Physics, Faculty of Sciences, University of Novi Sad, 21000 Novi Sad, Serbia; zeljka.cvejic@df.uns.ac.rs (Z.C.); resan.mirko@gmail.com (M.R.); 7 Faculty of Medicine of the Military Medical Academy, University of Defense, 11000 Belgrade, Serbia; 8 Medical Faculty, University of Belgrade, 11000 Belgrade, Serbia; bogdanresan@yahoo.com

**Keywords:** LASIK, femtosecond laser, mechanical microkeratome, high-order aberration, wavefront analysis, visual acuity

## Abstract

**Background/Objectives**: The technique used for flap creation in laser in situ keratomileusis (LASIK) may influence postoperative optical quality and visual outcomes. This prospective randomized paired-eye study compared higher-order aberrations (HOAs) and visual acuity outcomes following femtosecond laser-assisted versus mechanical microkeratome-assisted LASIK. **Materials and Methods**: Forty-four patients (88 eyes) underwent bilateral LASIK. In each patient, one eye was randomly assigned to high-frequency femtosecond laser flap creation (Femto LDV), and the fellow eye to mechanical microkeratome flap creation (Amadeus II). Inclusion criteria were stable refraction, central corneal thickness ≥ 520 µm, and normal corneal topography. HOAs were measured using Hartmann–Shack wavefront aberrometry over a 6 mm pupil diameter. Uncorrected and corrected distance visual acuity (UDVA and CDVA) were evaluated preoperatively and postoperatively at 1 day, 1 week, and 1, 3, and 6 months. **Results**: Both techniques induced significant postoperative changes in specific Zernike coefficients and an increase in total HOA root mean square (RMS) values (*p* < 0.05). A reduction in spherical aberration (Z4,0) was observed in both groups, while technique-specific changes were noted in individual aberration components including an increase in horizontal trefoil (Z3,3) in the femtosecond and a decrease in horizontal coma (Z5,1) in the microkeratome group. However, paired-eye comparisons revealed no statistically significant differences in total HOA six months postoperative. Despite comparable aberrometric outcomes, femtosecond-treated eyes demonstrated significantly better UDVA and CDVA at all postoperative time points (*p* < 0.05). **Conclusions**: Femtosecond laser-assisted and microkeratome-assisted LASIK resulted in comparable changes in higher-order aberrations, despite differing pattern in individual aberration components. The observed differences in visual acuity outcomes were not reflected in wavefront metrics, suggesting that postoperative visual performance may be influenced by factors.

## 1. Introduction

Laser in situ keratomileusis (LASIK) is one of the most widely performed refractive surgical procedures for correcting refractive errors such as myopia and hyperopia with and without a combination of astigmatisms and aberrations [1,2]. A critical step in LASIK is the creation of a corneal flap, which allows stromal ablation with an excimer laser to achieve the desired refractive correction [3].

The introduction of femtosecond laser technology significantly improved flap precision and reproducibility, resulting in greater predictability of flap thickness and morphology [4,5,6,7]. Comparative studies have demonstrated higher accuracy and reduced complication rates with femtosecond laser-assisted flap creation compared with mechanical microkeratomes [8,9,10].

One of the recognized consequences of corneal refractive surgery is the introduction of higher-order aberrations (HOAs), which may affect optical quality and cause visual symptoms such as glare, halos, and reduced contrast sensitivity [11,12,13]. Wavefront aberrations are commonly described using Zernike polynomials, and the magnitude of optical distortion is quantified by the root mean square (RMS) value [14].

The flap-creation technique may influence corneal biomechanics and anterior surface regularity, thereby affecting postoperative HOAs [15,16]. Previous studies comparing femtosecond and mechanical microkeratome LASIK have reported variable effects on optical quality [17,18,19,20]. However, many investigations used parallel-group designs, which are subject to inter-patient variability.

A paired-eye design allows intra-individual comparison, minimizing confounding factors related to corneal biomechanics and wound healing response.

Therefore, the present prospective randomized paired-eye study was designed to compare higher-order aberrations and visual outcomes following femtosecond laser-assisted versus mechanical microkeratome-assisted LASIK over a 6-month follow-up period.

We hypothesized that femtosecond laser-assisted flap creation may influence postoperative HOA profiles and visual outcomes compared with microkeratome flap creation.

## 2. Materials and Methods

### 2.1. Study Design and Participants

This prospective, randomized, paired-eye study was conducted at the ORASIS eye clinic in Reinach, Switzerland. Forty-four patients (88 eyes) aged 20–59 years undergoing bilateral LASIK were included. In each patient, one eye was randomly assigned to femtosecond laser-assisted flap creation (Femto LDV; Ziemer Ophthalmic System AG, Port, Switzerland), and the fellow eye to mechanical microkeratome-assisted flap creation (Amadeus II; Ziemer Ophthalmic System AG). Inclusion criteria were: age ≥ 20 years, stable refraction (±0.5 D change within 2 years), central corneal thickness ≥ 520 μm, normal corneal topographic without signs of ectatic disease, and expected postoperative residual stromal bed parameters within safety limits. Exclusion criteria included previous cataract or refractive surgery, abnormal corneal topographical, dry-eye syndrome, amblyopia, or other ocular pathology affecting visual outcomes. The study was adhered to the tenets of the Declaration of Helsinki and was approved by the local institutional review board (Kantonale Ethikkommission Aargau; protocol number 2006/11) and written informed consent was obtained from all participants. A post hoc power analysis based on the observed paired differences in HOA RMS was performed to estimate the achieved statistical sensitivity of the study. The included sample of 44 patients corresponded to approximately 80% power to detect a moderate effect size (Cohen’s d ≈ 0.5) at two-sided alpha level of 0.05 for paired comparisons.

### 2.2. Surgical Technique

All procedures were performed on the same day to minimize inter-day variability.

#### 2.2.1. Femtosecond Laser Group

The femtosecond laser group underwent surgery using the high-frequency femtosecond laser system FEMTO LDV Crystal Line (Ziemer Ophthalmic Systems, Port, Switzerland). This system employs a mode-locked, diode-pumped oscillator based on an ytterbium-doped yttrium aluminum garnet (Yb:YAG) laser crystal, operating at a wavelength of 1030 nm with a spectral bandwidth of 20–40 nm. Ytterbium (Yb), a rare earth element, provides a broader gain bandwidth compared to neodymium-doped lasers, enabling more efficient laser operation. The Yb:YAG crystal functions as a three-level laser system at 1030 nm. Due to partial photon reabsorption within the crystal, heat generation occurs, necessitating effective cooling. To address this, the laser utilizes thin-disk geometry for the gain medium, which enhances heat dissipation. The laser head incorporates a thin-disk Yb:YAG crystal [21] combined with a semiconductor saturable absorber mirror (SESAM) [22] enabling passive mode locking. Moreover, the laser system allows for pulse duration extension via passive mode locking by employing two distinct reflection angles within the thin disk. This design supports a power-scalable concept that broadens the laser’s applicability across various tissue types [23]. This design expands the laser’s applicability to various tissue types. Notably, the LDV Crystal Line is unique in cataract surgery due to its capability to operate either with applanation (direct contact with the cornea) or via a liquid interface, allowing versatile application such as in refractive corneal surgery. The pulse energy ranges from 50 nJ to 25 μJ with pulse durations of 200–350 femtoseconds (fs), adjusted according to the targeted tissue. The system features adaptive pulse management technology, enabling dynamic control of pulse energy—delivering higher energy at deeper ocular layers while minimizing energy at the surface to reduce collateral damage. Additionally, an adaptive optical system continuously optimizes laser spot focusing across different tissue planes. Consequently, the LDV Crystal Line supports a wide range of corneal surgical procedures, including LASIK flap creation, lamellar and penetrating keratoplasty, corneal and arcuate incisions, intracorneal ring segment implantation, intrastromal pocket creation, and cataract surgery steps such as customized lens fragmentation.

Thanks to its large numerical aperture, the LDV Crystal Line produces small, highly precise laser spots, which necessitate a high repetition rate in the MHz range. The laser spots overlap to create smooth, continuous cuts without residual tissue bridges. The tissue ablation proceeds in a peripheral-to-central direction. The treatment sequence begins with a circular peripheral incision with a target diameter of 9.5 mm. The treatment algorithm then involves creating a central corneal flap using a fast scan. This results in a gently declining topography at the periphery of the flap, merging into a central horizontal incision plane at a depth of 110 µm. This sequential approach yields optimal cutting quality. Additionally, the LDV Crystal Line is a fully mobile laser platform, easily deployable across different operating rooms and ready for use within 30 min [11,24].

The flaps were created using the Femto LDV Crystal Line femtosecond laser. A target flap diameter of 9.5 mm was programmed, with a superior hinge and a target thickness of 110 µm. During the cut, the cornea is flattened using a suction pressure of 600 mbar. This ensures a perfect reference plane for the cut. The cut flap is then mechanically mobilized and opened. The correction is performed using an excimer laser. The procedure concludes with irrigation of the interface space and repositioning of the flap.

#### 2.2.2. Mechanical Microkeratome Group

Flaps were created using Amadeus^®^ microkeratome (Ziemer Ophthalmic System AG, Port, Switzerland) with a target diameter of 9.5 mm and nasal hinge configuration. The intended flap thickness was 140 µm. The Amadeus microkeratome operated at an oscillation frequency of 11,000 rpm with a blade advanced rate of 1.6 mm/second. Stromal ablation was subsequently performed using the same excimer platform, followed by flap repositioning.

### 2.3. Wavefront Aberrometry

High order aberrations (HOA) were measured using Hartmann-Shack technique wavefront aberrometry (Zywave II, Bausch&Lomb incorporated Technolas 217p Zyoptic System, Bridgewater, NJ, USA). Measurements were obtained over a standardized 6 mm pupil diameter. The total HOA was quantified using the root-mean-square (RMS) of third- and higher-order Zernike coefficients.

### 2.4. Visual Acuity Assessment

Uncorrected distance visual acuity (UDVA) and corrected distance visual acuity (CDVA) were assessed preoperatively and postoperatively at 1 day, 1 week and 1, 3 and 6 months.

### 2.5. Statistical Analysis

Statistical analyses were performed using SPSS software (version 22; IBM Corp., Armonk, NY, USA). Given the paired-eye design, comparisons between femtosecond laser-treated and microkeratome-treated eyes were performed using paired statistical methods.

Normality of paired differences was evaluated using the Shapiro–Wilk test. Normally distributed variables were analyzed using paired-sample *t*-tests, whereas non-normally distributed variables were analyzed using the Wilcoxon signed-rank test.

The primary measure of the outcome was the inter-technique difference in total HOA RMS at 6 months postoperatively. Individual Zernike coefficients were analyzed as secondary, exploratory outcomes.

Changes in visual acuity over time within each treatment group were assessed using the Friedman test for repeated measures, followed by post hoc pairwise comparisons with adjustment for multiple testing when appropriate. A two-sided *p*-value ≤ 0.05 was considered statistically significant. All *p*-values were two-sided. In addition, a refractive vector analysis was performed according to the Alpins Method using manifest refractive astigmatism data. The analysis included target-induced astigmatism (TIA), surgically induced astigmatism (SIA), difference vector (DV), correction index (CI), index of success (IOS), flattening index (FI), magnitude of error, and angle of error.

In addition, a refractive vector analysis was performed according to the Alpins Method using manifest refractive astigmatism data [25,26]. The analysis included target-induced astigmatism (TIA), surgically induced astigmatism (SIA), difference vector (DV), correction index (CI), index of success (IOS), flattening index (FI), magnitude of error, and angle of error.

## 3. Results

### 3.1. Study Population and Baseline Characteristics

A total of 44 patients (88 eyes) were included. The mean age was 36.61 ± 8.36 years. Baseline refractive characteristics (sphere, cylinder, and spherical equivalent) were comparable between femtosecond laser and microkeratome-treated eyes (Table 1). Preoperative Zernike coefficients did not differ significantly between paired eyes (*p* > 0.05; Wilcoxon signed-rank test).

Preoperative Zernike coefficients were comparable between femtosecond laser-treated and microkeratome-treated eyes, with no statistically significant differences observed at baseline (Wilcoxon signed rank test; *p* > 0.05).

Paired-eye comparisons demonstrated that both femtosecond laser-assisted and microkeratome-assisted LASIK induced statistically significant postoperative changes in selected higher-order aberrations (Table 2 and Table 3).

Both techniques resulted in significant changes in quatrefoil (Z4,4) and a significant increase in total HOA RMS. A significant reduction in spherical aberration (Z4,0) was observed following both surgical approaches (*p* = 0.019 for the femtosecond group and *p* = 0.002 for the microkeratome group).

In the femtosecond laser group, LASIK resulted in a statistically significant increase in horizontal trefoil (Z3,3) (*p* = 0.017). In contrast, the microkeratome group demonstrated a significant decrease in horizontal coma (Z5,1) (*p* = 0.017), whereas changes in secondary astigmatism (Z4,2) did not reach statistical significance (*p* = 0.15).

### 3.2. Within-Group Changes in Higher-Order Aberrations (Preoperative vs. 6 Months)

Paired comparisons showed that both femtosecond laser and microkeratome flap creation were associated with significant postoperative changes in selected Zernike coefficients and with an increase in total HOA RMS at 6 months (Table 2 and Table 3).

In the femtosecond laser group, significant changes were observed in horizontal trefoil (Z3,3), spherical aberration (Z4,0), and quatrefoil (Z4,4) (all *p* < 0.05). Total HOA RMS increased from 0.40 ± 0.18 to 0.61 ± 0.31 (*p* < 0.001).

In the microkeratome group, significant changes were observed in spherical aberration (Z4,0), quatrefoil (Z4,4), and horizontal coma (Z5,1) (all *p* < 0.05). Secondary astigmatism (Z4,2) did not reach statistical significance (*p* = 0.15). Total HOA RMS increased from 0.41 ± 0.17 to 0.57 ± 0.24 (*p* < 0.001).

Paired-eye comparisons demonstrated no statistically significant differences in postoperative higher-order aberrations between femtosecond laser-treated and microkeratome-treated eyes (Table 4).

Although significant within-group changes were observed in selected Zernike coefficients, direct paired-eye comparisons revealed no statistically significant differences in postoperative HOA profiles between femtosecond laser-assisted and microkeratome-assisted flap creation.

**Figure 1:**
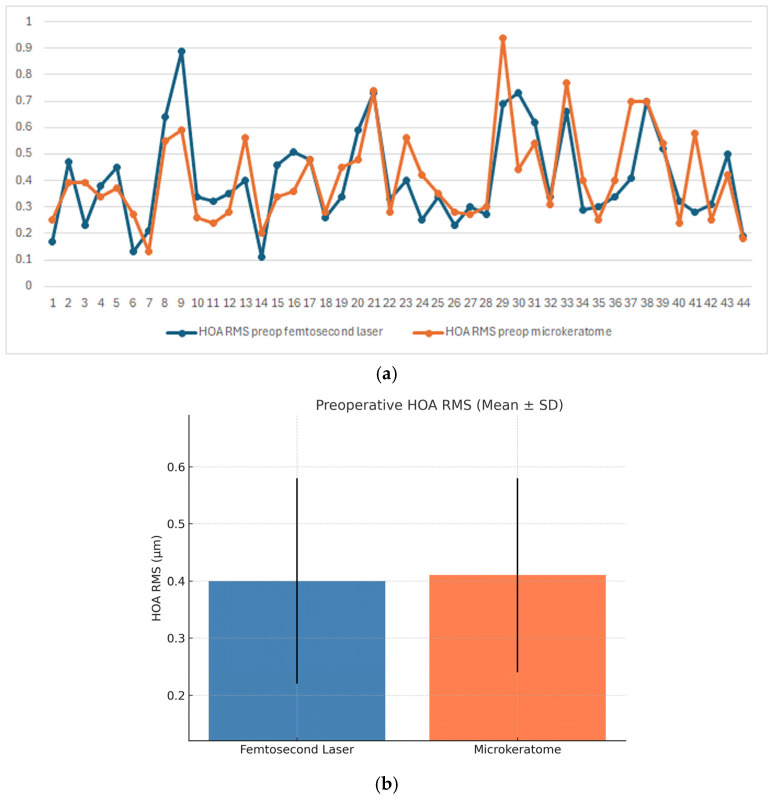
(**a**) Preoperative higher-order aberration root mean square (HOA RMS) comparison between femtosecond laser and mechanical microkeratome groups: Individual preoperative total HOA RMS values for paired eyes treated with femtosecond laser and microkeratome. (**b**) Mean preoperative total HOA RMS values with standard deviation (mean ± standard deviation) showing comparable baseline aberration levels between the two groups (femtosecond laser: 0.40 ± 0.18 µm and microkeratome: 0.41 ± 0.17 µm).

**Figure 2:**
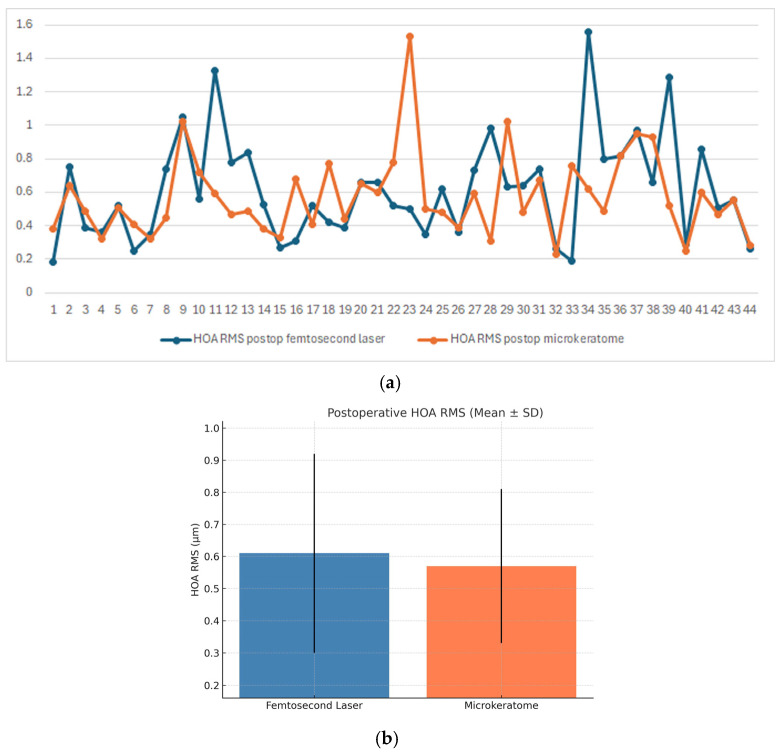
(**a**) Postoperative higher-order aberration root mean square (HOA RMS) at 6 months following LASIK. Individual postoperative HOA RMS values in femtosecond laser-treated and microkeratome-treated eyes. (**b**) Mean postoperative HOA RMS values with standard deviation (mean ± SD) at 6 months (femtosecond laser: 0.61 ± 0.31 µm; microkeratome: 0.57 ± 0.24 µm).

**Figure 3:**
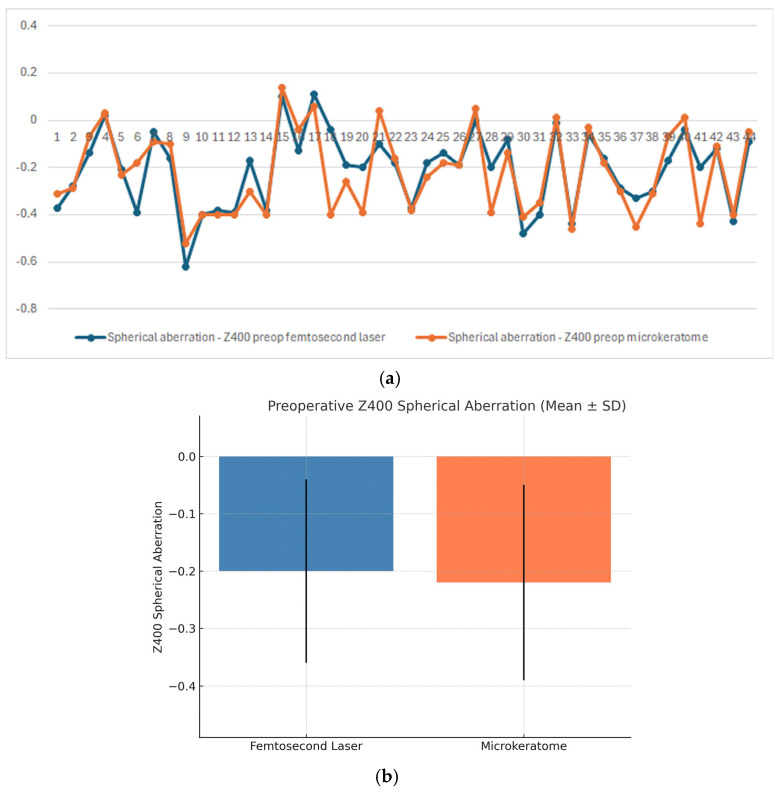
(**a**) Preoperative spherical aberration (Z4,0) comparison between femtosecond laser and mechanical microkeratome groups. Individual preoperative Z4,0 spherical aberration values in paired eyes. (**b**) Mean preoperative Z4,0 spherical aberration values with standard deviation (mean ± SD), demonstrating similar baseline optical characteristics between groups (femtosecond laser: −0.20 ± 0.16 µm; microkeratome: −0.22 ± 0.17 µm).

**Figure 4:**
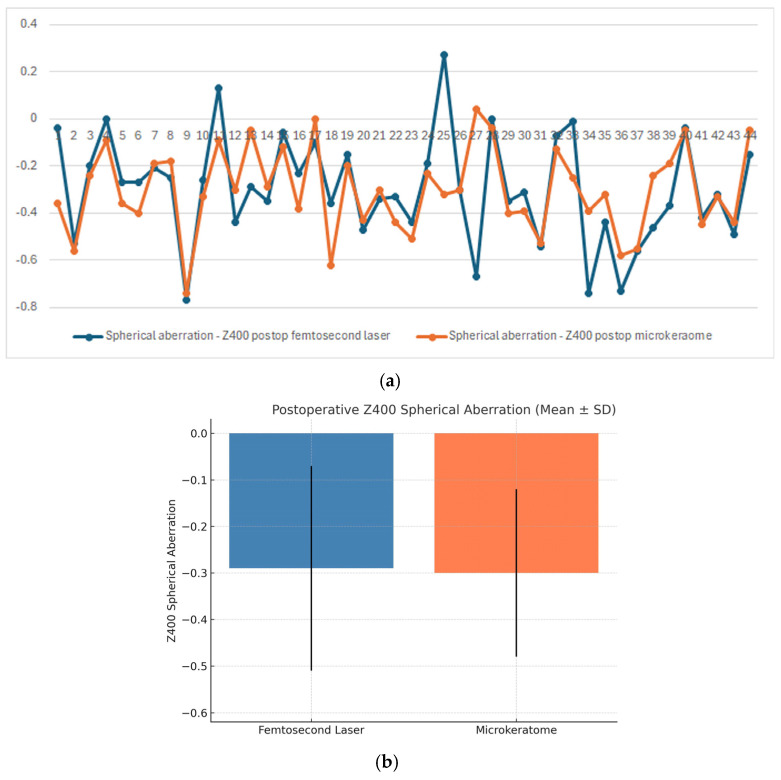
(**a**) Postoperative spherical aberration (Z4,0) at 6 months after LASIK. Individual postoperative Z4,0 spherical aberration values in femtosecond laser and microkeratome groups. (**b**) Mean postoperative Z4,0 spherical aberration values with standard deviation (mean ± SD) at 6 months (femtosecond laser: −0.29 ± 0.22 µm; microkeratome: −0.30 ± 0.18 µm).

Preoperative and postoperative comparisons of total HOA RMS between femtosecond laser-treated and microkeratome-treated eyes are presented in Figure 1 and Figure 2, respectively. Preoperative and postoperative spherical aberration (Z4,0) values are illustrated in Figure 3 and Figure 4.

### 3.3. Uncorrected Distance Visual Acuity (UCVA) and Corrected Distance Visual Acuity (CDVA)

The Friedman test demonstrated statistically significant in both uncorrected distance visual acuity (UDVA) and corrected distance visual acuity (CDVA) over time in the femtosecond laser group when postoperative measurements were compared with preoperative values (*p* < 0.05; Table 5). Similarly, significant improvements in UDVA were observed in the microkeratome group during postoperative follow-up (Table 6).

Paired-eye comparisons demonstrated no significant difference in UDVA between treatment groups preoperatively. However, femtosecond laser-treated eyes achieved significantly better postoperative UDVA compared with microkeratome-treated eyes at all postoperative follow-up visits (Table 7).

Paired-eye analysis demonstrated no significant difference in CDVA between treatment groups preoperatively. However, femtosecond laser-treated eyes showed significantly better postoperative CDVA compared with microkeratome-treated eyes at all postoperative follow-up time points (Table 8).

**Figure 5:**
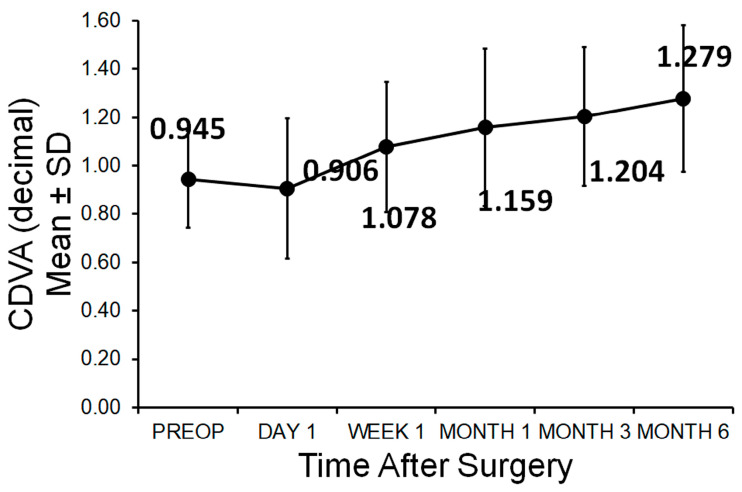
Changes in corrected distance visual acuity (CDVA) over the 6-month postoperative follow-up in the femtosecond laser group. Values represent mean ± standard deviation.

In the femtosecond laser group, the CDVA was 0.945 ± 0.19 preoperatively, 0.906 ± 0.29 1 day postoperatively, 1.078 ± 0.27 after 1 week, 1.158 ± 0.33 after 1 month, 1.204 ± 0.29 after 3 months and 1.279 ± 0.30 after 6 months (Figure 5).

**Figure 6:**
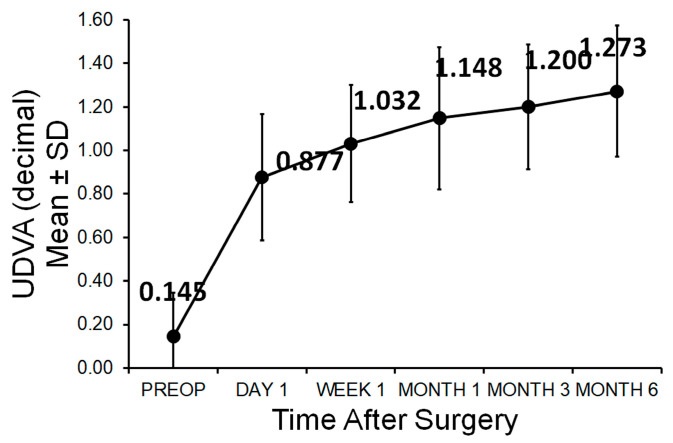
Changes in uncorrected distance visual acuity (UDVA) during the 6-month postoperative follow-up in the femtosecond laser group. Values represent mean ± standard deviation.

The UDVA was 0.145 ± 0.11 preoperatively, 0.887 ± 0.30 1 day postoperatively, 1.032 ± 0.30 after 1 week, 1.148 ± 0.34 after 1 month, 1.200 ± 0.29 after 3 months and 1.273 ± 0.31 after 6 months (Figure 6).

**Figure 7:**
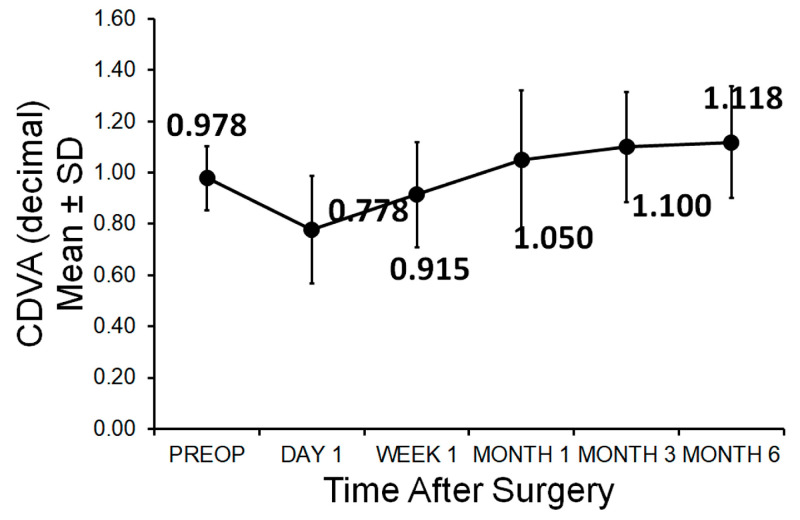
Changes in corrected distance visual acuity (CDVA) during the 6-month postoperative follow-up in the microkeratome group. Values represent mean ± standard deviation.

In the microkeratome group, the CDVA was changed from 0.978 ± 0.13 preoperatively to 0.771 ± 0.21 at postoperatively day 1, 0.915 ± 0.21 at 1 week, 1.050 ± 0.27 at 1 month, 1.101 ± 0.21 at 3 months and 1.118 ± 0.22 at 6 months (Figure 7).

**Figure 8:**
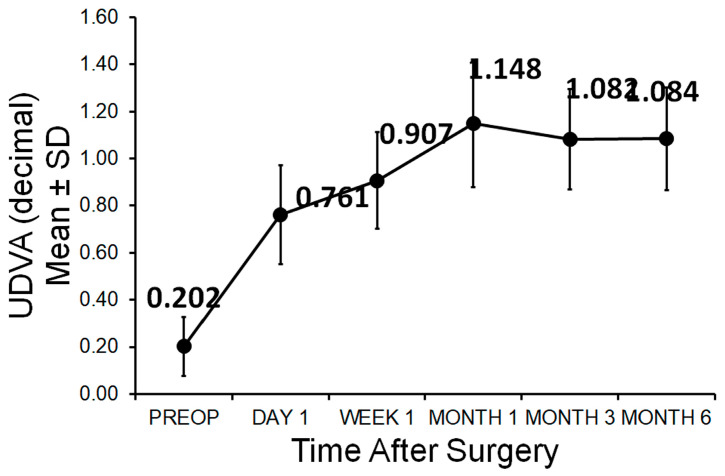
Changes in corrected distance visual acuity (UDVA) during the 6-month postoperative follow-up in the microkeratome group. Values represent mean ± standard deviation.

In the microkeratome group, mean UDVA improved from 0.202 ± 0.20 preoperatively to 0.761 ± 0.22 at postoperative day 1, 0.907 ± 0.21 at week 1, 1.031 ± 0.28 at month 1, 1.082 ± 0.23 at month 3, and 1.084 ± 0.21 at month 6 (Figure 8).

In addition to the Friedman test results already presented, we performed a Wilcoxon signed-rank test to compare postoperative visual acuity between the femtosecond laser and microkeratome-treated eyes at each time point. These comparisons revealed statistically significant differences in UDVA and CDVA in favor of the femtosecond laser group at 1 day, 1 week, 1 month, 3 months, and 6 months postoperatively (*p* < 0.05 at each time point; full data presented in Table 9).

### 3.4. Refractive Vector Analysis (Alpins Method)

A refractive vector analysis according to the Alpins Method was performed using manifest refractive astigmatism data [25,26]. Both treatment groups demonstrated excellent astigmatic correction.

The mean correction index was close to unity in both groups (1.03 ± 0.14 in the femtosecond laser group and 1.08 ± 0.14 in the microkeratome group), indicating accurate astigmatic correction. Difference vector values were low in both groups (0.06 ± 0.14 D), reflecting minimal residual astigmatism. Similarly, angle of error and magnitude of error remained close to zero, suggesting high accuracy of astigmatic correction.

**Figure 9:**
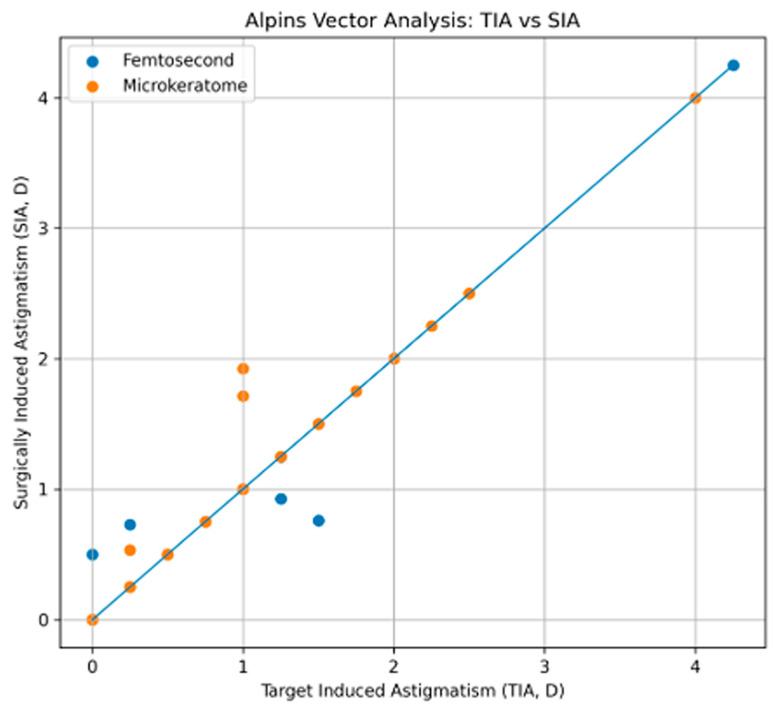
Relationship between target-induced astigmatism (TIA) and surgically induced astigmatism (SIA) according to the Alpins Method in femtosecond laser-assisted and microkeratome-assisted LASIK. The diagonal line represents perfect agreement between intended and achieved astigmatic correction.

**Figure 10:**
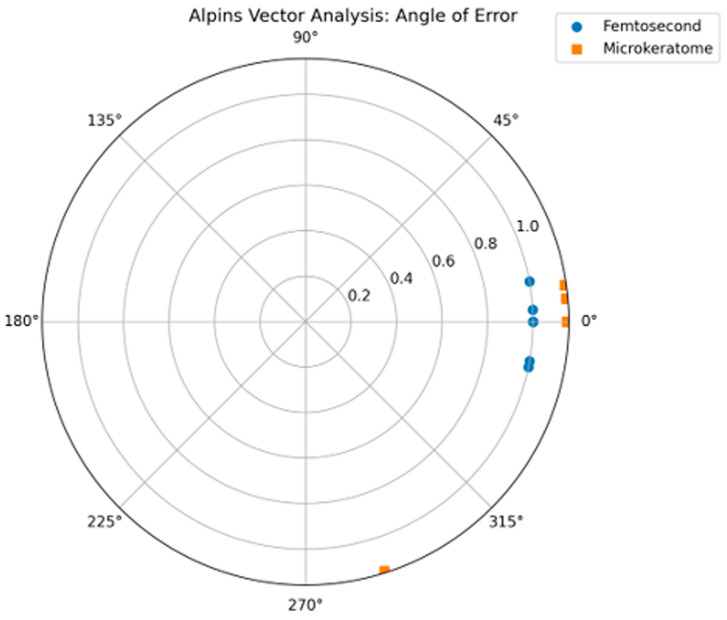
Polar distribution of angle of error according to the Alpins Method following femtosecond laser-assisted and microkeratome-assisted LASIK. Values clustered around 0° indicate accurate alignment of the achieved astigmatic correction.

Figure 9 illustrates the relationship between target-induced astigmatism (TIA) and surgically induced astigmatism (SIA). In both groups, data points were closely distributed around the line of identity, indicating good agreement between intended and achieved astigmatic correction. Figure 10 demonstrates the polar distribution of angle of error, with values clustered around zero degrees in both groups.

Overall, the Alpins vector analysis did not reveal clinically meaningful differences between femtosecond laser-assisted and microkeratome-assisted LASIK and demonstrated comparable refractive astigmatic outcomes (Table 10).

## 4. Discussion

The present prospective randomized paired-eye study evaluated the effects of femtosecond-assisted and mechanical microkeratome-assisted LASIK flap creation on higher-order aberrations and visual outcomes over 6-month follow-up period. The main finding of this study is that although both surgical techniques induced comparable postoperative changes in higher-order aberrations, femtosecond laser-treated eyes achieved consistently superior visual outcomes. These results suggest that postoperative visual performance following LASIK may not depend solely on the magnitude of induced optical aberration.

Both flap-creation techniques resulted in a significant increase in total HOA RMS values at 6 months postoperatively. This finding is consistent with numerous previous studies reporting increased higher-order aberrations following LASIK regardless of the method used for flap creation [27,28,29,30,31]. In addition, significant postoperative changes were observed in specific Zernike coefficients, particularly horizontal trefoil (Z3,3), spherical aberration (Z4,0), and quatrefoil (Z4,4), confirming that corneal reshaping during LASIK inevitably alters higher-order optical properties of the eye.

Interestingly, in contrast to the majority of previously published studies describing an increase in spherical aberration after LASIK, the present study demonstrated a statistically significant reduction in spherical aberration (Z4,0) in both treatment groups. One possible explanation for this discrepancy lies in methodological difference between studies. In the present analysis, spherical aberration was evaluated using signed Zernike coefficients, allowing assessment of the direction of change. Many earlier investigations reported RMS-derived values, which are always positive and therefore do not distinguish between increases and decreases in aberration magnitude. Consequently, direct comparison between studies should be interpreted with caution [31,32,33,34]. Differences in ablation profiles, preoperative refractive error distribution, and postoperative corneal biomechanical responses may further contribute to variability in reported outcomes.

Based on the higher precision and reproducibility of femtosecond laser flap creation, it has frequently been hypothesized that femtosecond-assisted LASIK induced fewer higher-order aberrations than mechanical microkeratome [5,26,29]. However, an important finding of the present study is that postoperative higher-order aberrations did not differ significantly between femtosecond laser-treated and microkeratome-treated eyes. This observation agrees with the systematic review and meta-analysis by Chen et al. [27], which reported no significant differences in postoperative HOA induction between the two flap-creation techniques. Similar conclusions have also been reported in comparative clinical studies demonstrating comparable optical outcomes despite technological differences between devices [32]. These findings suggest that while femtosecond lasers offer greater precision in flap creation, this advantage does not necessarily translate into measurable differences in postoperative HOA profiles. The impact on overall HOA induction may vary depending on patient-specific factors and study methodologies. The additional refractive vector analysis performed according to the Alpins Method-yielded findings consistent with the wavefront analysis. Parameters describing astigmatic correction accuracy, including correction index, difference vector, angle of error, and index of success, demonstrated comparable outcomes between groups. Furthermore, the relationship between target-induced and surgically induced astigmatism showed excellent agreement for both flap-creation techniques.

Therefore, neither wavefront analysis nor refractive vector analysis identified substantial differences in postoperative optical performance between femtosecond laser-assisted and microkeratome-assisted LASIK. The superior visual acuity outcomes observed in the femtosecond laser group cannot be readily explained by differences in postoperative astigmatic correction accuracy alone.

Despite comparable postoperative HOA profiles, femtosecond laser-treated eyes achieved significantly better postoperative UDVA and CDVA throughout the follow-up period. This observation represents one of the most noteworthy findings of the present study and suggests that visual performance following LASIK may not be fully explained by conventional wavefront-derived HOA measurements alone.

The present study was not designed to identify the mechanisms responsible for this discrepancy. Consequently, the observed visual acuity advantage associated with femtosecond laser flap creation cannot be directly attributed to factors such as corneal biomechanics, flap morphology, stromal bed characteristics, epithelial remodeling, or other structural parameters, as these variables were not evaluated in the current investigation. Therefore, the present findings should not be interpreted as evidence supporting any specific mechanism underlying the observed differences in visual outcomes.

The present study has several limitations. First, although the paired-eye design improves internal validity, the sample size remains moderate and may limit the detection of small inter-technique differences in individual aberration components. Second, higher-order aberrations were evaluated using a single wavefront measurement system, and additional corneal biomechanical, epithelial remodeling, or corneal topographic analyses were not performed. Consequently, the mechanisms underlying the observed differences in visual acuity could not be fully explored. Finally, longer follow-up periods could further clarify the long-term optical and functional stability following flap creation using different technologies.

From a clinical perspective, both flap-creation techniques demonstrated favorable safety and efficacy profiles. The absence of significant differences in postoperative HOA measurements suggests that both methods provide comparable optical outcomes. At the same time, the consistently superior visual acuity observed in the femtosecond laser group indicates that factors beyond the measured HOA parameters may contribute to functional postoperative vision.

The paired-eye design of the present study provides robust comparative evidence that both flap-creation techniques produce comparable higher-order aberration outcomes, while femtosecond laser-assisted LASIK was associated with superior visual acuity results. Further investigations incorporating corneal biomechanical, epithelial, and topographic analyses are required to better understand the factors underlying this difference [33,34].

The additional refractive vector analysis performed according to the Alpins Method [25,26] yielded findings consistent with the wavefront analysis. Parameters describing astigmatic correction accuracy, including correction index, difference vector, angle of error, and index of success, demonstrated comparable outcomes between groups. Furthermore, the relationship between target-induced and surgically induced astigmatism showed excellent agreement for both flap-creation techniques.

Therefore, neither wavefront analysis nor refractive vector analysis identified substantial differences in postoperative optical performance between femtosecond laser-assisted and microkeratome-assisted LASIK.

The mechanism underlying the superior visual acuity outcomes observed in the femtosecond laser group remains uncertain. Visual acuity may be influenced by factors that are not fully captured by total HOA RMS values or refractive vector analysis, including light scatter, epithelial remodeling, flap-interface regularity, contrast sensitivity, and corneal biomechanical responses. Since these parameters were not evaluated in the present study, no causal explanation can be established based on the available data. Future investigations incorporating these additional measures may help clarify the observed discrepancy between objective optical metrics and visual acuity outcomes.

## 5. Conclusions

Both femtosecond laser-assisted and microkeratome-assisted LASIK resulted in significant postoperative improvements in visual acuity and induced comparable changes in higher-order aberrations. No statistically significant differences in postoperative HOA profiles were observed between the two flap-creation techniques.

Both surgical approaches were associated with an increase in total HOA RMS values, whereas a significant reduction in spherical aberration (Z4,0) was observed in both groups. These findings indicate that the method of flap creation has limited influence on postoperative HOA outcomes as assessed by Hartmann–Shack wavefront aberrometry.

Despite comparable postoperative HOA profiles, femtosecond laser-treated eyes achieved significantly better postoperative UDVA and CDVA throughout the follow-up period. This finding suggests that visual performance following LASIK may not be fully explained by conventional wavefront-derived HOA measurements alone.

The paired-eye design of the present study provides robust comparative evidence that both flap-creation techniques produce comparable optical aberration outcomes, while femtosecond laser-assisted LASIK was associated with superior visual acuity results. Further studies incorporating corneal biomechanical, epithelial, topographic, and additional visual quality assessments are required to clarify the factors underlying this discrepancy and to better characterize postoperative visual performance following LASIK.

Despite comparable postoperative HOA profiles and comparable refractive vector analysis outcomes, femtosecond laser-treated eyes achieved significantly better postoperative UDVA and CDVA throughout follow-up. The mechanism underlying this discrepancy remains uncertain and warrants further investigation.

## Figures and Tables

**Table 1:** Baseline characteristics.

Parameter	Mechanical Microkeratome	Femtosecond Laser
Eyes (*n*)	44	44
Patients (*n*)	44	44
Sex (male/female)	27/17	27/17
Age * (years)	36.61 ± 8.36	36.61 ± 8.36
Sphere * (D)	−2.31 ± 1.96	−2.42 ± 2.18
Cylinder * (D)	−0.82 ± 0.82	−0.89 ± 0.75
Spherical equivalent *	−2.72 ± 1.96	−2.88 ± 2.15

* Values are presented as mean ± standard deviation.

**Table 2:** Preoperative versus 6-month postoperative changes in higher-order aberration in femtosecond laser group (n = 44).

Parameter	Preoperative (Mean ± SD)	6 Months (Mean ± SD)	*p*-Value
Z3,3 Horizontal trefoil	0.0007 ± 0.12	0.06 ± 0.16	0.017
Z4,0 Spherical aberration	−0.20 ± 0.16	−0.29 ± 0.22	0.019
Z4,4 Quatrefoil	−0.004 ± 0.07	0.03 ± 0.12	0.040
Total HOA RMS (6 mm pupil)	0.40 ± 0.18	0.61 ± 0.31	<0.001

**Table 3:** Preoperative versus 6-month postoperative changes in higher-order aberration in microkeratome group (n = 44).

Parameter	Preoperative (Mean ± SD)	6 Months (Mean ± SD)	*p*-Value
Z4,0 Spherical aberration	−0.22 ± 0.17	−0.30 ± 0.18	0.002
Z4,2 Secondary astigmatism	−0.0082 ± 0.08	0.03 ± 0.11	0.150 *
Z4,4 Quatrefoil	0.0057 ± 0.09	0.06 ± 0.11	0.009
Z5,1 Horizontal coma	0.007 ± 0.03	−0.01 ± 0.06	0.017
Total HOA RMS (6 mm pupil)	0.41 ± 0.17	0.57 ± 0.24	<0.001

* Not statistically significant.

**Table 4:** Paired-eye comparison of postoperative higher-order aberrations at 6 months between femtosecond laser and microkeratome-treated eyes.

Parameter	Mean Paired Difference (Femto − Micro)	Standard Deviation	*p*-Value
Vertical coma (Z3,1)	−0.041	0.356	0.440
Horizontal trefoil (Z3,3)	0.040	0.285	0.349
Spherical aberration (Z4,0)	0.004	0.191	0.876
Secondary astigmatism (Z4,2)	−0.010	0.115	0.543
Quatrefoil (Z4,4)	−0.030	0.157	0.206
Total HOA RMS (6 mm pupil)	0.037	0.343	0.470

**Table 5:** Changes in UDVA and CDVA over time in femtosecond laser group (Friedman post hoc analysis).

Comparison	UDVA *p*-Value	CDVA *p*-Value
Preop vs. Day 1	<0.001	>0.99
Preop vs. Week 1	<0.001	0.29
Preop vs. Month 1	<0.001	<0.001
Preop vs. Month 3	<0.001	<0.001
Preop vs. Month 6	<0.001	<0.001

**Table 6:** Changes in UDVA and CDVA over time in microkeratome group (Friedman post hoc analysis).

Comparison	UDVA *p* Value	CDVA *p*-Value
Preop vs. Day 1	0.004	<0.001
Preop vs. Week 1	<0.001	>0.99
Preop vs. Month 1	<0.001	>0.99
Preop vs. Month 3	<0.001	0.038
Preop vs. Month 6	<0.001	0.006

**Table 7:** Paired-eye comparison of UDVA between femtosecond laser-treated and microkeratome-treated eyes at each follow-up visit (Wilcoxon signed-rank test).

Time Point	Z Value	*p*-Value
Preoperative	−1.200	0.23
1 day postoperative	−2.139	0.02
1 week postoperative	−2.029	0.03
1 month postoperative	−2.184	0.02
3 month postoperative	−2.398	0.01
6 month postoperative	−3.229	<0.001

**Table 8:** Paired-eye comparison of postoperative CDVA between femtosecond laser-treated and microkeratome-treated eyes (Wilcoxon signed-rank test).

Time Point	Z Value	*p*-Value
Preoperative	−1.266	0.20
1 day postoperative	−2.648	<0.001
1 week postoperative	−2.955	<0.001
1 month postoperative	−2.277	0.02
3 month postoperative	−2.484	0.01
6 month postoperative	−2.906	<0.001

**Table 9:** Paired-eye comparison of postoperative visual acuity between femtosecond laser-treated and microkeratome-treated eyes (Wilcoxon signed-rank test).

Time Point	Outcome	Z-Value	*p*-Value
1 day postoperative	UDVA	−2.193	0.02
	CDVA	−2.648	<0.001
1 week postoperative	UDVA	−2.092	0.03
	CDVA	−2.955	<0.001
1 month postoperative	UDVA	−2.184	0.02
	CDVA	−2.277	0.02
3 month postoperative	UDVA	−2.398	0.01
	CDVA	−2.484	0.01
6 month postoperative	UDVA	−3.229	<0.001
	CDVA	−2.906	<0.001

**Table 10:** Alpine vector analysis parameters.

Parameter	Femtosecond Group	Microkeratome Group
TIA (D)	0.88 ± 0.52	0.78 ± 0.52
SIA (D)	0.88 ± 0.48	0.82 ± 0.48
DV (D)	0.06 ± 0.14	0.06 ± 0.14
Angle of Error (°)	−0.22 ± 9.75	−1.70 ± 9.75
Magnitude of Error (D)	0.00 ± 0.11	0.04 ± 0.11
Correction Index	1.03 ± 0.14	1.08 ± 0.14
Index of Success	0.09 ± 0.23	0.14 ± 0.23
Flattening Index	1.02 ± 0.20	0.97 ± 0.20

## Data Availability

The data presented in this study are available on request from the first and the corresponding authors, in particular the datasets are archived at the treating clinics. The data are not publicly available as they contain information that could compromise the privacy of the participants.

## Abbreviations

The following abbreviations are used in this manuscript:

LASIK	laser in situ keratomileusis
HOA	high order aberration
UDVA	uncorrected distance visual acuity
CDVA	corrected distance visual acuity
Postop.	postoperative
RMS	root mean square

## References

[1] Kymionis G.D., Tsiklis N.S., Astyrakakis N., Pallikaris A.I., Panagopoulou S.I., Pallikaris I.G. (2007). Eleven-Year Follow-up of Laser in Situ Keratomileusis. J. Cataract. Refract. Surg..

[2] Du T.T., Fan V.C., Asbell P.A. (2007). Conductive Keratoplasty. Curr. Opin. Ophthalmol..

[3] Ratkay-Traub I., Ferincz I.E., Juhasz T., Kurtz R.M., Krueger R.R. (2003). First Clinical Results with the Femtosecond Neodynium-Glass Laser in Refractive Surgery. J. Refract. Surg. Thorofare.

[4] Farjo A.A., Sugar A., Schallhorn S.C., Majmudar P.A., Tanzer D.J., Trattler W.B., Cason J.B., Donaldson K.E., Kymionis G.D. (2013). Femtosecond Lasers for LASIK Flap Creation: A Report by the American Academy of Ophthalmology. Ophthalmology.

[5] Xia L.-K., Yu J., Chai G.-R., Wang D., Li Y. (2015). Comparison of the Femtosecond Laser and Mechanical Microkeratome for Flap Cutting in LASIK. Int. J. Ophthalmol..

[6] Tham V.M., Maloney R.K. (2000). Microkeratome Complications of Laser in Situ Keratomileusis. Ophthalmology.

[7] Hoffmann S., Krummenauer F., Tehrani M., Dick H.B. (2003). Impact of Head Advance and Oscillation Rate on the Flap Parameter: A Comparison of Two Microkeratomes. Graefe’s Arch. Clin. Exp. Ophthalmol..

[8] Lackerbauer C.-A., Grueterich M., Kojetinsky C., Ulbig M., Kollias A. (2009). Customizing the Amadeus II Microkeratome: Evaluation of Cut Quality with Various Settings Using Electron Microscopy. Eur. J. Ophthalmol..

[9] Shah R. (2019). History and Results; Indications and Contraindications of SMILE Compared With LASIK. Asia-Pac. J. Ophthalmol..

[10] Pajic B., Pajic-Eggspuehler B., Rathjen C., Resan M., Cvejic Z. (2021). Why Use Ultrashort Pulses in Ophthalmology and Which Factors Affect Cut Quality. Medicina.

[11] Pajic B., Vastardis I., Pajic-Eggspuehler B., Gatzioufas Z., Hafezi F. (2014). Femtosecond Laser versus Mechanical Microkeratome-Assisted Flap Creation for LASIK: A Prospective, Randomized, Paired-Eye Study. Clin. Ophthalmol..

[12] Murakami Y., Manche E.E. (2011). Comparison of Intraoperative Subtraction Pachymetry and Postoperative Anterior Segment Optical Coherence Tomography of Laser in Situ Keratomileusis Flaps. J. Cataract. Refract. Surg..

[13] Santhiago M.R., Kara-Junior N., Waring G.O. (2014). Microkeratome versus Femtosecond Flaps: Accuracy and Complications. Curr. Opin. Ophthalmol..

[14] Kanclerz P., Khoramnia R. (2021). Flap Thickness and the Risk of Complications in Mechanical Microkeratome and Femtosecond Laser In Situ Keratomileusis: A Literature Review and Statistical Analysis. Diagnostics.

[15] Bühren J., Kohnen T. (2006). Factors Affecting the Change in Lower-Order and Higher-Order Aberrations after Wavefront-Guided Laser in Situ Keratomileusis for Myopia with the Zyoptix 3.1 System. J. Cataract. Refract. Surg..

[16] Miret J.J., Rojas E., Camps V.J., Garcia C., Caballero M.T., Martín B., Chipont E. (2022). Understanding the Real Effect of the High-Order Aberrations after Myopic Femto-Lasik. Optics.

[17] Oshika T., Klyce S.D., Applegate R.A., Howland H.C., El Danasoury M.A. (1999). Comparison of Corneal Wavefront Aberrations after Photorefractive Keratectomy and Laser in Situ Keratomileusis. Am. J. Ophthalmol..

[18] Lee J.M., Lee D.J., Jung W.J., Park W.C. (2008). Comparison between Anterior Corneal Aberration and Ocular Aberration in Laser Refractive Surgery. Korean J. Ophthalmol. KJO.

[19] Sharma M., Wachler B.S.B., Chan C.C.K. (2007). Higher Order Aberrations and Relative Risk of Symptoms after LASIK. J. Refract. Surg. Thorofare.

[20] Salmon T.O., van de Pol C. (2006). Normal-Eye Zernike Coefficients and Root-Mean-Square Wavefront Errors. J. Cataract. Refract. Surg..

[21] Giesen A., Hügel H., Voss A., Witting K., Brauch U., Opower H. (1994). Scalable concept for diode-pumped high-power solid-state lasers. Appl. Phys. B.

[22] Keller U., Miller D.A.B., Boyd D.G., Chiu T.H., Ferguson J.F., Asom M.T. (1992). Solid-state low-loss intracavity saturable absorber for Nd:YLF lasers: An antiresonant semiconductor Fabry-Perot saturable absorber. Opt. Lett..

[23] Brunner F., Paschotta R., Aus der Au J., Spuehler J., Morier-Genoud F. (2001). Widely turnable pulses durations from a passively mode-locked thin-disk Yb: YAG laser. Opt. Lett..

[24] Pajic B., Cvejic Z., Pajic-Eggspuehler B. (2017). Cataract Surgery Performed by High Frequency LDV Z8 Femtosecond Laser: Safety, Efficacy, and Its Physical Properties. Sensors.

[25] Alpins N.A. (1993). A new method of analyzing vectors for changes in astigmatism. J. Cataract. Refract. Surg..

[26] Alpins N.A. (2001). Practical applications of the Alpins method for planning and analyzing astigmatic outcomes. J. Cataract. Refract. Surg..

[27] Chen S., Feng Y., Stojanovic A., Jankov M.R., Wang Q. (2012). IntraLase Femtosecond Laser vs Mechanical Microkeratomes in LASIK for Myopia: A Systematic Review and Meta-Analysis. J. Refract. Surg. Thorofare.

[28] Yvon C., Archer T.J., Gobbe M., Reinstein D.Z. (2015). Comparison of Higher-Order Aberration Induction between Manual Microkeratome and Femtosecond Laser Flap Creation. J. Refract. Surg. Thorofare.

[29] Lim T., Yang S., Kim M., Tchah H. (2006). Comparison of the IntraLase Femtosecond Laser and Mechanical Microkeratome for Laser in Situ Keratomileusis. Am. J. Ophthalmol..

[30] Chan A., Ou J., Manche E.E. (2008). Comparison of the Femtosecond Laser and Mechanical Keratome for Laser in Situ Keratomileusis. Arch. Ophthalmol..

[31] Montés-Micó R., Rodríguez-Galietero A., Alió J.L. (2007). Femtosecond Laser versus Mechanical Keratome LASIK for Myopia. Ophthalmology.

[32] Hasimoto A.R., Gomes M.F.B., de Siqueira M.A.V., Moreira H. (2013). Femtosecond laser versus mechanical microkeratome for LASIK flap creation. Arq. Bras. Oftalmol..

[33] Guber I., Moetteli L., Magnin L., Majo F. (2013). Moving from a Mechanical Microkeratome to a Femtosecond Laser for LASIK to Correct Astigmatic Patients: Clinical Outcomes of a Retrospective, Consecutive, Comparative Study. Klin. Monatsbl. Augenheilkd..

[34] Kanellopoulos A.J., Asimellis G. (2013). Long-Term Bladeless LASIK Outcomes with the FS200 Femtosecond and EX500 Excimer Laser Workstation: The Refractive Suite. Clin. Ophthalmol..

